# Viral load dynamics among adults receiving HIV care in rural North-Eastern South Africa, 2015–2020: insights from a population-based record linkage study

**DOI:** 10.3389/fpubh.2025.1551847

**Published:** 2025-05-07

**Authors:** Armstrong Dzomba, Francesc Xavier Gomez-Olive, Jean Bashingwa, Morelearnings Sibanda, Belinda Njiro, Kathleen Kahn, Daniel Ohene-Kwofie, Chodziwadziwa Kabudula

**Affiliations:** Medical Research Council/Wits Rural Public Health and Health Transitions Research Unit, Johannesburg, South Africa

**Keywords:** sustained viral suppression, treatment failure, viral rebound, viral response, antiretroviral therapy, HIV/AIDS

## Abstract

**Introduction:**

The Joint United Nations Programme on HIV/AIDS set ambitious-but-reachable targets to have 95% of HIV-positive people diagnosed, 95% on ART, and 95% virally suppressed by 2030. To address the latter, post-2016, South Africa’s HIV treatment guidelines aimed to deliver maximal and durable viral load (VL) suppression through extensive antiretroviral therapy (ART) scale-up. Yet, standard suppression one-off measurement conceals viral response trajectories with high onward transmission potential for HIV patients on lifelong treatment. We investigated the dynamics of periodic VL patterns and associated socio-demographic factors in rural north-eastern South Africa using data from adults receiving HIV care in healthcare facilities within the Agincourt Health and Demographic Surveillance System (HDSS).

**Methods:**

We extracted two person-identified VL measurements collected 9-15 months apart per individual yearly between 2015 and 2020 from the Agincourt HDSS Hospital-Clinic-Linkage system for 7 493 HIV patients. Sankey diagrams were used to describe VL flows within and across the suppressed and unsuppressed statuses over each year. We classified temporal VL responses into four profiles: (i) Sustained suppression, (ii) achieved suppression, (iii) viral rebound, (iv) virologic failure. Additionally, mixed-effects multinomial logistic regression models were utilised to examine the odds of covariates factors for varied VL trajectories.

**Results:**

The proportion of individuals remaining virally suppressed increased steadily from 84% in 2015 to 86% in 2016, with the highest prevalence of 88% sustained for three consecutive years, from 2017 through 2019, and then dropped slightly in 2020 to 87%. However, 2-3% of initially virally suppressed rebounded annually, while ~5% experience treatment failure. The likelihood of achieving viral suppression was high among men, those aged 15-24 years and 25-34 years however, these groups were less likely to have sustained viral suppression and more likely to experience virologic failure and rebounding.

**Conclusions:**

Temporal VL metrics are needed to effectively track progress towards reaching high and sustained HIV suppression potential in HIV hyperendemic settings. Thus, optimising the assessment of targeted interventions and identification of left-behind groups such as those younger, men, unmarried and poorer HIV patients to improve individual and population health outcomes.

## Introduction

Since 2015, in South Africa HIV Viral Load (VL) has been the recommended single most important biomarker for monitoring treatment success (persistently undetectable VL below 1,000 copies/mL) and early identification of treatment failure (VL > 1,000 copies/mL on consecutive measurements) among adults (1). In late 2016, universal test and treat (UTT) was adopted as the main strategy to deliver maximal and durable suppression of VL for patients through scaled-up of antiretroviral therapy (ART) (2, 3). However, the status of the suppressed or unsuppressed dichotomy as the standard-of-care for tracking response to ART has been challenged in recent studies (4, 5), suggesting that the launch of 95–95-95 targets should be accompanied by an expansion of monitoring tools to characterize HIV viral response trajectories more comprehensively. The Joint United Nations Programme on HIV/AIDS set ambitious-but-reachable targets to have 95% of HIV-positive people diagnosed, 95% on ART, and 95% virally suppressed by 2030 (6, 7). The success of implementing UTT and achieving a high rate of viral suppression in a country with a high HIV burden would be both desirable and demonstrative of the potential for continued progress toward reversing the AIDS epidemic by halting new infections, which is the goal of the 95–95-95 targets. Overall, by 2017, South Africa was on track toward achieving the third UNAIDS 95% target (8). However, subpopulation differences are concealed when socio-demographic and spatial characteristics are not accounted for (9). Large key subpopulations are yet to accrue the suppressive benefit of ART, such as men (10–12), those aged 15–24 years (13), adults between 25 to 49 in rural areas, and residents in farming communities (14, 15) Motivated by the inadequate methods to operationalize viral response and identify at-risk groups, Shiau et al. (4) used a time-dependent approach to characterize virologic response patterns among infants initiated on ART by designating them into the ‘virologic success’, ‘virologic rebound’ and ‘virologic failure’ groups since baseline status. Application of this approach to adult patients has begun elsewhere in sub-Saharan Africa. A retrospective study conducted in Ghana found that maintaining viral suppression and rebounding was associated with drug adherence and baseline VL, among other factors (5). However, long-term viral response data remains fragmented by focusing on binary outcomes (15) smaller samples (16), longer inter-survey periods (17) single health facility (17) or multi-centre comparative designs for decentralized service delivery (DSD) vs. clinics (18, 19) studies.

In line with the World Health Organisation (WHO) mantra ‘living no one behind’, there is a need for granular, time-based viral response metrics to deepen our understanding of VL patterns and associated covariate information for people engaged in HIV care in health facilities during a time when HIV services needed strengthening. As the proportion of PLHIV who rebound from viral suppression after previous episodes of being suppressed or unsuppressed increases (4, 5), it is critical to capture these explicit and expected patient VL trajectories, which better reflect actual experiences of PLHIV. Unfortunately, most current HIV care delivery models fail to properly account for the differential and temporal dimensions of viral responses (4) given that sequential VL measurements produce trends that are rendered invisible with single time-point data. To fill this critical data gap, it is crucial to investigate the socio-demographic predictors of diverse viral responses for HIV simultaneously for improved intervention target setting. The presence of a large subset of long-term infectious patients [i.e., those rebounding, 13% in South Africa (17) or treatment failure groups] within the treated population poses onward transmission and drug resistance risk and may jeopardize efforts to control the epidemic (20, 21). Adolescent boys and young men (ABYM) constitute a large proportion of those unsuppressed (8, 22). This further reduces the chance of adolescent girls and young women (AGYW) to achieve suppression goals despite being the focus of large-scale efforts to improve HIV prevention and treatment services (23), such as Determined, Resilient, Empowered, AIDS-free, Mentored and Safe (DREAMS) project in sub-Saharan Africa, where the epidemic continues to be driven by heterosexual contact. An updated and iterative viral load monitoring can be an essential approach to achieve the 95–95-95 objective and timely optimization of measuring progress toward reaching HIV suppression potential in high prevalence settings assess targeted interventions and identify modifiable risk factors for both individual and population health gains. In this study we assess viral load dynamics and associated socio-demographic risk factors among adults receiving HIV care in a rural setting in Mpumalanga province, South Africa during the period 2016–2020.

## Materials and methods

### Study design

We conducted a retrospective study of patients enrolled in ART program in primary healthcare facilities within the Agincourt Health and Demographic Surveillance System (HDSS) study area in the Bushbuckridge sub-district of Mpumalanga Province between January 2015 and December 2020.

### Study setting and population

The Agincourt HDSS covers 31 villages spread across an area of 450 km^2^ in rural north-eastern South Africa in the Bushbuckridge sub-district of Ehlanzeni District, Mpumalanga Province. The population under surveillance, estimated at approximately 116,000 individuals, is largely Xitsonga-speaking, with one-third being self-settled former Mozambican refugees and their descendants who arrived in the area in the 1980s. There are two public health centres and seven clinics within the area, with two district hospitals 25–60 km apart (24). High HIV characterises the setting, with a prevalence peak of 45% among older adults aged 35–39 years, rates above 15 and 10% persist until age 70 for men, and women, respectively (25). Given that most other rural settings in South Africa, ART has been provided within primary care clinics since 2010 through nurse-led, devolved, public-sector ART programs, and treatment coverage expanded in line with changes in national guidelines over time. In December 2014, recommendations for ART initiation included all pregnant or breastfeeding women and any patient with a CD4 count <500/lL (26). Eligibility criteria further extended in 2016 to enrol all persons with confirmed HIV positive test results (27). For HIV treatment cascade outcomes, amongst adults aged ≥40 in the study area, 63% of those living with HIV were on ART, of whom 72% had viral suppression in 2015 (28). Our analytic sample comprised of HIV-positive individuals who were on ART for at least six months before the first VL measurement in 2015. Between 2015 and 2020, 19,651 HIV positive patients were captured by the Clinic Link database and were assessed for initial eligibility into our study, of whom, 9,755 were excluded either for failing to meet the age and calendar time for initiating ART inclusion criteria as shown in Figure 1. With a cohort selection base of 9,896, a further 1,559 were not considered for having no consecutive VL readings 9 to 15 months apart post initiation. This group encapsulates patient loss to follow-up given the widely spaced laboratory footprint of some patients on ART. Our analytic population for the statistical analysis featured 7,493 patients who had ≥2 periodical VL trajectory measures.

**Figure 1:**
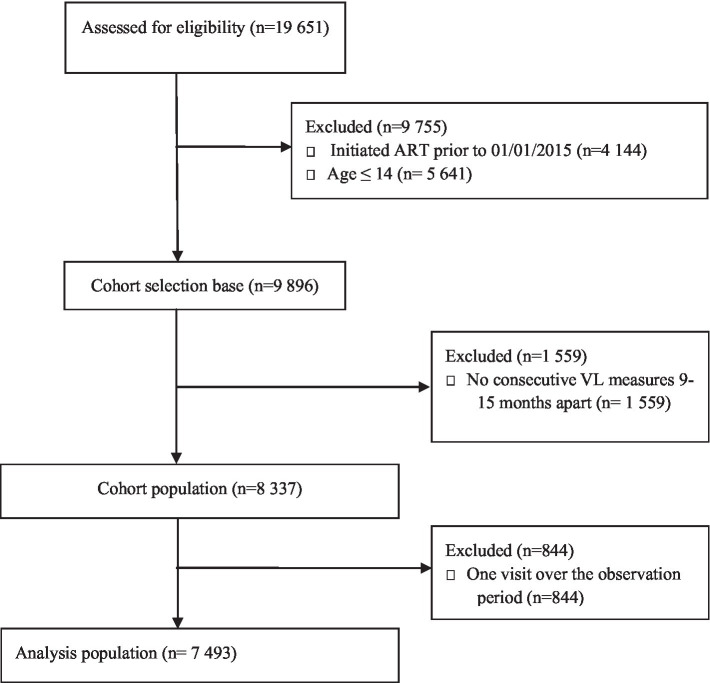
CONSORT flow diagram detailing selection of participants in the analysis population.

### Data sources

Data for the study arose from the Agincourt HDSS-Clinic Link System. As has been described elsewhere (28, 29), this is an ongoing longitudinal data platform that captures and stores patient identifiers, demographic and clinical information collected from consenting patients seeking HIV-specific services or chronic care in all seven publicly funded healthcare facilities within the Agincourt HDSS study area. Since 2014, trained Data typists have been stationed at each of the health facilities, daily seeking the consent of patients to capture and link their demographic and clinical data to the Agincourt HDSS data which contains routinely updated detailed information on vital events (births, deaths and migrations) and complementary socio-economic indicators such as marital status, education, ethnicity, and household socio-economic status (24). On an ongoing basis, the Data typists update clinical information into the Clinic Link system as patients return for services. All analytic data are anonymised before sharing with third-party users. Ethical approval was obtained from both University of the Witwatersrand Human Sciences Research Ethics Committee M151162.

### Outcome measurement

We measured HIV viral suppression status using the WHO definition (1) with HIV viral suppression being VL < 1,000 copies/mL and non-suppression being VL ≥ 1,000 copies/mL. Individuals who had been on ART for less than six months were excluded from the study. We used a 6-month time-lag duration on ART, which is the minimum time required to determine viral response according to the 2015 national treatment guidelines (30), because ART initiation does not result in immediate viral suppression.

As suppression status may vary over time, we categorized patients into four viral response groups based on clinically valid viral endpoints (virologic success, achieving suppression, rebound, and failure). We compared consecutive VL measurements recorded in a nine to 15-month follow-up period against the first VL measurements per year^1^ for each patient. From the baseline virological status, we defined virologic success as sustaining viral suppression through nine to 15 months. Achieving suppression was defined as being virologically unsuppressed at the first VL measurement, with subsequent VL suppression at nine to 15 months later. Viral rebound was defined as being virally unsuppressed at nine to 15 months after being virally suppressed at the baseline VL measurement (1). Viral failure was defined as never having achieved viral suppression both at baseline VL measurement and between nine to 15 months later.

### Exposure variables and covariates

Time-invariant factors of interest included were: level of education (none or primary, 0–7 years; secondary, 8–12 years; tertiary, >12 years); marital status (married, unmarried); household wealth (using quintiles of the first component identified by principal components analysis of household assets and characteristics); residency status (permanent residents, temporary residents); ethnicity (formerly Mozambican, bonafide South African); and history of pregnancy. Additional covariates included age at baseline and sex. The information for all the covariates came from the Agincourt HDSS.

### Statistical analysis

We conducted three distinct analyses to characterize the HIV viral dynamics. Firstly, for each calendar year, we calculated the total number of; HIV patients, clinic visits attended, including median visits per patient and associated interquartile ranges, and counts of HIV patients on ART, those on ART and have at least one VL test and the prevalence of viral suppression based on the latest VL reading. We matched two person-identified VL measurements for each patient: the first VL reading of the calendar year (*t_1_*) and then 9–15 months later (*t_2_*) between 2015 and 2020 for those engaged in HIV care in study area clinics. Then, we calculated percentages for patients’ viral suppression status (suppressed; unsuppressed) at *t_1_* and viral outcomes derived from changes within or across the initial viral suppression status at *t_2_*, categorized into four patient groups; viral success, achieving suppression, rebounding, and viral failure. Using Sankey diagrams, we evaluated HIV viral transitions between *t_1_* and *t_2_* per year to visually represent the underlying patterns, trends, and intensity of flows of HIV patients’ status from either virally suppressed or unsuppressed at one-time point to remaining suppressed, achieving suppression, rebounding, and treatment failure at the other successive observation periods between 2015 and 2020. Sankey diagrams are generally used to perform visual analysis of multidimensional data to map out processes and flows in a system (31) - in our case, the thickness of the lines is proportional to the flow quantity. Lastly, we fitted mixed-effects multinomial logistic regression models to examine the association between various socio-demographic factors and HIV VL trajectories. The covariate factors included in our models were preselected for their significance in previous analyses (10–13) An essential feature of this model is accounting for the repeated VL outcomes for each individual over the six-observation time-points, i.e., from 2015 to 2020. This model accommodates multiple random effects and allows for a general form of model covariates while being applicable to nominal response data (32).

## Results

Between 2015 and 2020, 9,896 HIV-positive adults aged 15 years and older participated in the HDSS Clinic-Link system. Of these participants, on average 8,161 (86.9%) had initiated HIV treatment. The median age (IQR) of the study participants at the first clinic visit was 39 (15–96) years, and 74.9% were female (not shown).

Table 1 shows that HIV-positive patients contributed on average approximately 60,000 clinic visits per year in the HDSS Clinic-Link platform, with a minimum of 44,317 visits in 2015 and a peak of 71,438 in 2019. The median number of clinic visits per year for each patient ranged between five and six visits. The proportion of HIV-positive patients on ART was 70.7% in 2015 and increased to 85.6% in 2016 and reached above 90% from 2018 through 2020. Overall, the percentage of patients with at least one VL measurement among those receiving ART per year declined steadily, with the maximum being 69.7% (5,477 of 7,862) and a minimum of 45% (5,249 of 11,665) in 2020. Based on these first readings the prevalence of HIV viral suppression increased gradually from 86% in 2015 to 89% in 2016; from 2017 onwards, the percentage of virally suppressed cases was consistently above 90%.

**Table 1:** Annual number of HIV positive cases, corresponding clinic visits, number of participants on ART and prevalence of viral suppression from 2015 to 2020.

	Number of persons HIV-positive	No of clinic visits	Median no. of clinic visits per patient	Total on ART	Total on ART with at least one viral load test	Proportion virally suppressed (based on one reading)
Year	*N*	*n*	*n* (IQR)		*n*	% (95% CI)
2015	8,331	44,317	5 (4)	6,222 (74.7)	3,588 (57.7)	2,727, 85.5 (84.2–86.7)
2016	9,405	57,217	6 (4)	7,862 (83.6)	5,477 (69.7)	4,890, 89.8 (88.9–90.6)
2017	10,643	59,058	5 (3)	9,446 (88.8)	6,177 (65.4)	5,611, 90.8 (90.1–91.5)
2018	11,365	61,551	5 (4)	10,342 (91.0)	6,999 (67.7)	6,407, 91.5 (83.7–86.3)
2019	12,147	71,438	6 (3)	11,185 (92.1)	6,662 (59.6)	6,105, 91.6 (90.9–92.3)
2020	12,495	68,886	6 (3)	11,665 (93.4)	5,249 (45.0)	4,953, 91.2 (90.4–92.0)

IQR, inter-quartile range; CI, confidence interval.

Table 1 presents description of the number of patients living with HIV per each calendar year, the number of clinic visits attended by HIV patients, the median number of clinic visits per HIV patient and interquartile range (IQR), the total number of ART and the percentage of virally suppressed cases between 2015 to 2020.

Although patients with two consecutive viral load measurements (9–15 intervals) represented 97% of the 3,588 total patients with viral load measurements in 2015, they represented approximately 70% of the eligible patient population during any follow-on observation period beyond 2015. The VL transitions flowchart for each year are shown in Figure 2 which isa Sankey diagram showing HIV viral dynamics and transitions by calendar year from 2015 to 2020.

**Figure 1:**
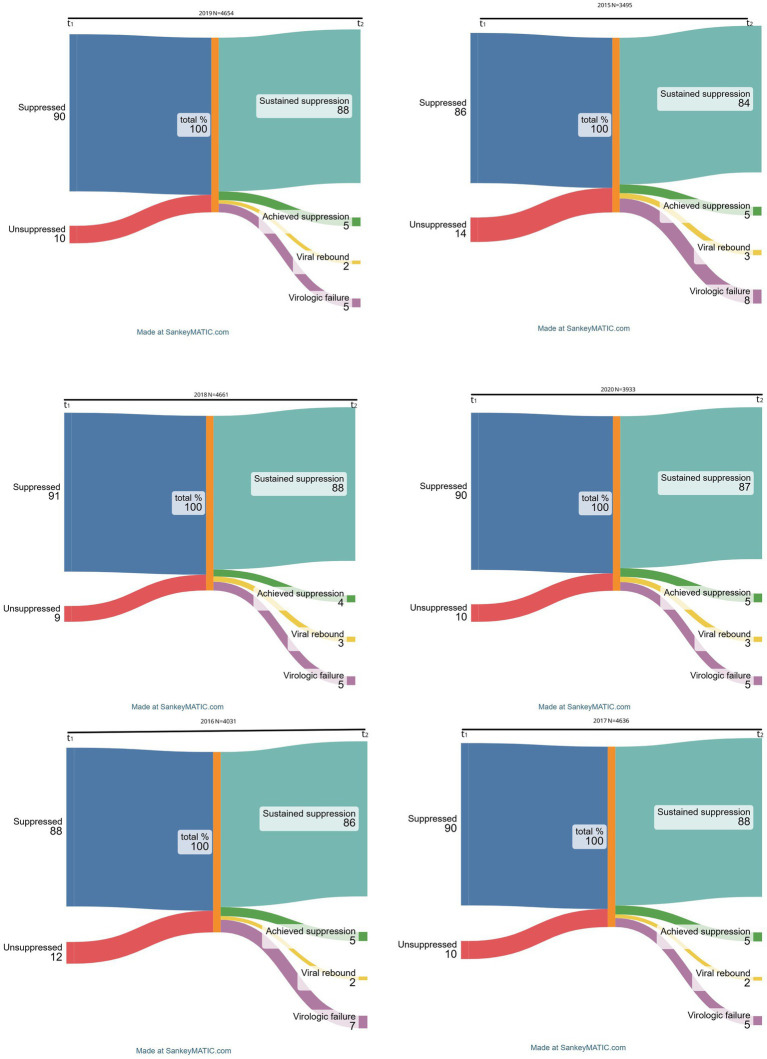
Trends, patterns, and intensity of flows for viral response trajectories between 2015-2020.

Most of the HIV patients had sustained viral suppression of above 80% between 2015 and 2020. In fact, the percentage of those remaining virally suppressed increased steadily from 84% in 2015 to 86% in 2016, with the highest prevalence of 88% being maintained for three consecutive years, from 2017 through 2019, and then dropped slightly in 2020 to 87%. Those attaining suppression, i.e., previously virally unsuppressed but achieving viral suppression at the second consecutive VL measurement, contributed 5% of the transitions across all the years except for 2018 when prevalence declined to 4%. On the other hand, the proportion of participants with treatment failure, i.e., persistently unsuppressed VL, decreased over time, with a maximum of 8% in 2015, lowered to 7% in 2016 and remained unchanged at 5% from 2017 onwards. Lastly, ‘viral rebounds’ averaged between 2 and 3% since 2015, remaining at the maximum of 3% in the most recent observation period in 2020.

In our mixed-effects multinomial logistic regression models (*N* = 9,765) in Table 2, several socio-demographic factors were significantly associated with the four VL outcome profiles (sustained viral suppression, achieving suppression, viral rebound, and virologic failure). Compared to women, men had lower odds of sustained viral suppression (aOR = 0.72, 95% CI: 0.67–0.78). Moreover, the odds of sustained suppression increased with age; patients aged 15–24 years (aOR = 0.20; 95% CI: 0.17–0.23), 25–34 years (aOR = 0.45; 95% CI: 0.40–0.51), 35–44 years (aOR = 0.68; 95% CI: 0.60–0.76), and 45–54 years (aOR = 0.83; 95% CI: 0.72–0.94) compared to those aged 55 years and above had lower odds. Increased odds of virologic success were also associated with being historically Mozambican (aOR = 1.29; 95% CI: 1.15–1.45) compared to South African: and being treated in 2017 (aOR = 1.39; 95% C: 1.17–1.65), 2018 (aOR = 1.33; 95% CI: 1.12–1.57), 2019 (aOR = 1.26; 95% CI: 1.06–1.49) and 2020 (aOR = 1.28; 95% CI: 1.07–1.53) compared to 2015.

**Table 2:** Mixed effects models for associations between socio-demographic and health factors and HIV viral responses (sustained suppression, achieving suppression, virologic failure, and rebounding) among adults in rural Mpumalanga, South Africa (2015–2020) *N* = 7,493.

Variable	Sustained suppression		Achieve suppression		Viral rebound		Virologic failure	
	OR (95% CI)	aOR (95% CI)	OR (95% CI)	aOR (95% CI)	OR (95% CI)	aOR (95% CI)	OR (95% CI)	aOR (95% CI)
Sex: [Female]								
Male	0.72 (0.67–0.78)**	0.63 (0.56–0.71)**	1.14 (0.99–1.31)*	1.26 (1.04–1.53)**	1.25 (1.04–1.48)**	1.22 (0.96–1.55)	1.57 (1.40–1.76)**	1.88 (1.60–2.21)**
Age categories: [55y+]								
15-24y	0.20 (0.17–0.23)**	0.17 (0.13–0.21)**	3.39 (2.64–4.35)**	3.36 (2.30–4.90)**	3.10 (2.26–4.24)**	3.54 (2.18–5.73)**	5.33 (4.36–6.53)**	6.79 (4.85–9.50)**
25-34y	0.45 (0.40–0.51)**	0.39 (0.31–0.49)**	2.44 (2.01–2.98)**	2.32 (1.64–3.27)**	1.88 (1.46–2.42)**	1.96 (1.25–3.08)**	1.87 (1.57–2.23)**	2.59 (1.87–3.59)**
35-44y	0.68 (0.60–0.76)**	0.62 (0.50–0.77)*	1.39 (1.14–1.70)**	1.31 (0.93–1.84)	1.44 (1.13–1.85)**	1.40 (0.90–2.16)	1.47 (1.24–1.75)**	1.86 (1.35–2.54)**
45-54y	0.83 (0.72–0.94)*	0.80 (0.64–1.00)	1.33 (1.07–1.65)*	1.14 (0.80–1.63)	1.09 (0.82–1.45)	1.22 (0.77–1.93)	1.15 (0.94–1.39)	1.30 (0.92–1.82)
Marital status: [Married]								
Unmarried	1.24 (1.13–1.35)**	0.90 (0.81–1.01)	0.89 (0.77–1.02)	1.01 (0.85–1.21)	0.88 (0.73–1.06)	0.96 (0.77–1.20)	0.75 (0.66–0.86)**	1.24 (1.06–1.46)*
Highest education: [Tertiary]								
Primary	0.82 (0.63–1.08)	1.00 (0.72–1.40)	0.98 (0.65–1.48)	0.99 (0.58–1.71)	1.11 (0.64–1.93)	0.85 (0.44–1.63)	1.49 (0.97–2.27)	1.08 (0.66–1.77)
Secondary	0.85 (0.65–1.11)	1.12 (0.80–1.52)	1.02 (0.68–1.53)	0.96 (0.57–1.62)	1.07 (0.62–1.83)	0.85 (0.45–1.57)	1.36 (0.90–2.06)	0.92 (0.57–1.48)
Household wealth quintile: [Middle 20%]								
Bottom 20%	0.75 (0.65–0.85)**	0.86 (0.72–1.01)	1.17 (0.95–1.46)	1.00 (0.76–1.31)	1.18 (0.90–1.55)	1.10 (0.79–1.53)	1.49 (1.23–1.82)**	1.33 (1.03–1.71)*
Lower 20%	0.90 (0.78–1.03)	0.96 (0.81–1.13)	1.08 (0.87–1.34)	0.93 (0.71–1.22)	0.94 (0.71–1.25)	0.90 (0.64–1.26)	1.21 (0.99–1.49)	1.24 (0.98–1.59)
Higher 20%	0.94 (0.82–1.08)	0.89 (0.75–1.05)	1.02 (0.82–1.27)	1.09 (0.84–1.42)	0.84 (0.63–1.12)	0.85 (0.60–1.19)	1.22 (0.99–1.49)	1.29 (1.01–1.65)*
Top 20%	0.86 (0.75–1.00)	0.85 (0.72–1.01)	1.12 (0.90–1.40)	1.13 (0.87–1.46)	0.88 (0.66–1.17)	0.96 (0.69–1.34)	1.33 (1.09–1.62)*	1.29 (0.73–1.27)
Residency status: [Permanent resident]								
Temporary migrant	0.93 (0.81–1.07)	1.08 (0.91–1.29)	1.04 (0.83–1.30)	0.89 (0.68–1.18)	1.27 (0.96–1.66)	1.17 (0.85–1.62)	0.99 (0.81–1.22)	0.85 (0.67–1.10)
Refugee status: [South African]								
Formerly Mozambican	1.30 (1.20–1.40)**	1.29 (1.15–1.45)**	0.77 (0.68–0.87)**	0.72 (0.60–0.86)**	0.81 (0.69–0.96)**	0.73 (0.58–0.91)**	0.80 (0.71–0.89)	0.90 (0.76–1.06)
History of pregnancy: [No]								
Yes	0.88 (0.74–1.04)	0.83 (0.68–1.02)	1.12 (0.86–1.47)	1.14 (0.83–1.56)	1.10 (0.77–1.57)	1.06 (0.69–1.61)	1.14 (0.90–1.45)	1.28 (0.96–1.71)
Calendar year: [2015]								
2016	1.14 (1.00–1.29)**	1.13 (0.96–1.34)	0.96 (0.79–1.17)	0.94 (0.71–1.24)	0.87 (0.65–1.17)	0.87 (0.60–1.26)	0.85 (0.71–1.01)	0.88 (0.70–1.12)
2017	1.42 (1.26–1.62)**	1.39 (1.17–1.65)**	0.80 (0.66–0.98)*	0.91 (0.69–1.19)	0.85 (0.64–1.13)	0.72 (0.49–1.04)	0.63 (0.53–0.75)**	0.65 (0.51–0.83)**
2018	1.41 (1.24–1.60)**	1.33 (1.12–1.57)**	0.68 (0.55–0.83)**	0.76 (0.57–1.01)	1.08 (0.82–1.41)	1.01 (0.71–1.42)	0.66 (0.55–0.78)**	0.70 (0.55–0.89)**
2019	1.38 (1.21–1.56)**	1.26 (1.06–1.49)**	0.82 (0.67–0.99)*	1.00 (0.76–1.31)	1.05 (0.80–1.37)	0.96 (0.68–1.37)	0.60 (0.51–0.72)**	0.64 (0.50–0.81)**
2020	1.34 (1.18–1.52)**	1.28 (1.07–1.53)**	0.82 (0.67–1.01)	0.86 (0.65–1.15)	1.17 (0.89–1.55)	1.11 (0.78–1.59)	0.60 (0.50–0.72)**	0.64 (0.50–0.82)**

OR, odds ratio; aOR, adjusted odds ratio; CI, confidence interval; reference category in [brackets]; *p < 0.05; **p < 0.01.

Sex, age, and refugee status were independently associated with achieving viral suppression. Men compared to women (aOR = 1.26; 95% CI: 1.04–1.53), and those aged 15–24 years (aOR = 3.36; 95% CI: 2.30–4.90) compared to 55 years and above had significantly higher odds of achieving viral suppression. Being formerly Mozambican (aOR = 0.72; 95% CI: 0.60–0.86) was associated with a lower risk of achieving viral suppression.

Patients aged 15–24 years (aOR = 3.54; 95% CI: 2.18–5.73) and 25–34 years (aOR = 1.96, 95% CI: 1.25–3.08) had significantly higher odds of viral rebounding compared to those aged 55 years and above while formerly Mozambican refugees (aOR = 0.73, 95% CI: 0.58–0.91) had lower odds of viral rebounding compared to historically South Africans. Men compared to women (aOR = 1.88; 95% CI: 1.60–2.21), those aged 15–24 years (aOR = 6.79; 95% CI: 4.85–9.50), 25–34 years (aOR = 2.59; 95% CI: 1.87–3.59), and 35–44 years (aOR = 1.86; 95% CI: 1.35–2.54) compared to 55 years and above, and those from bottom 20% poorer households compared to middle 20% wealthier households had significantly higher risk of virologic failure. Additionally, being unmarried was associated with an increased risk of virologic failure risk (aOR = 1.24; 95% CI: 1.06–1.46).

## Discussion

Drawing from a large adult rural community sample in South Africa, this study examined HIV viral load trajectories and explored associated factors, producing three sets of results. First, the prevalence of viral suppression from 86 to 91% between 2015 and 2020. Second, we found reductions among unsuppressed HIV patients from 14% in 2015 to 10% in 2020, although ~2–3% rebound and 5% experience treatment failure every year. Further, we found that men and those younger than 45 years were associated with high odds of achieving viral suppression, virologic failure and rebounding, simultaneously, these groups also had lower odds of sustained viral suppression. Lastly, being unmarried and residence in a low SES household was associated with increased odds of virologic failure. This association among men, those younger, unmarried, and deprived - often characteristic of displaced populations, as echoed elsewhere (11, 14, 33) accounts for failure to achieve viral suppression. Nevertheless, we provide estimates of patients with intermittent viremia during an era of ART, i.e., rebounding and virologic failure, groups which are usually concealed in one-time measures such viral suppression status. This study illustrates the importance of conducting repeated viral load measurements for patients and highlights the complexity in viral load dynamics by identifying oscillations between suppression and non-suppression during treatment in some patients to better reflect the actual risk of onward transmission. The success of current interventions depends on massive and durable reductions in the unsuppressed and often left-behind populations, which is critical to achieving significant HIV suppression potential to halt new HIV transmissions by 2030.

We found routine VL suppression coverage increasing among patients on ART given programmatic conditions from 2015 to 2020 in rural communities in South Africa. In South Africa, UTT which expanded access to ART for patients regardless of clinical or immunological eligibility, was implemented late from 2016 onwards. Until 2016, viral suppression prevalence was below 90% and reached the 90% threshold from 2017 onwards, thus making our results comparable with a recent nationally representative study in South Africa (8). Although Marinda et al. (8) study was based on a 2017 population-based survey, our multi-year clinic-based study encompassed the corresponding year and prevalence matching national estimates. We show high viral suppression beyond 2017, signaling early progress for the country being on track toward achieving the UNAIDS 95–95-95 targets by 2030. The upward trajectory possibly reflects the successful implementation of the HIV care and treatment landscape in South Africa, which scaled-up protocols for monitoring patients with an elevated VL, supported downstream HIV services such as decentralized VL testing, increased testing modalities, trained healthcare workers in the collection, active-tracing, and utilization of VL test results (9, 33). Given the improved pharmacokinetics of newer ART regimens (i.e., dolutegravir-based in 2019) over therapies administered in the earlier treatment era may have increased the proportion virally suppressed patients overall (30, 34). However, access to VL testing coverage remains generally low despite service expansion into low-and middle-income countries after 2015, specifically among under-represented subpopulations, including men (11). In our study, men comprised only 25% of the total sample presenting for scheduled VL testing, highlighting the underlying healthcare utilization challenges which effective and acceptable interventions for men living with HIV (MLWH) can resolve (35). Further, the current analysis found that greater than 50% of HIV patients eligible for routine VL testing had no results. This does not only potentially highlight the impact COVID-19 (i.e., restricted movement, social distancing) on HIV services (36) but underrepresents population groups missing visits in 2020 in this study. Efforts to optimize care for men and strengthen health systems are essential in sub-Saharan Africa, where heterosexual contact remains the predominant route of transmission of HIV infection, as previous regional interventions largely targeted women and weaker pandemic resilience in southern Africa (22, 35, 37).

Overall, our HDSS Clinic-Link data demonstrated reductions in unsuppressed population levels following the expansion of ART programs, but residual groups with transmissible HIV remain important. Approximately 5% remain unsuppressed, and up to 3% oscillate from suppression to unsuppressed over the six-time points, including two observation periods when ART coverage was limited. This viral response scenario is problematic from a public health perspective: untreated individuals will remain infectious for longer periods and pose a higher risk of transmitting HIV infection to their uninfected sexual partner. Despite the inconsistency, previous studies have generally attributed socio-demographic, behavioral, and virologic factors to failure to achieve viral suppression; this includes young age, increased number of sexual partners, residential instability and longer residence in a higher HIV transmission setting, and drug resistance in South African rural and peri-urban communities (9). Our granular description of treatment outcomes helps to effectively identify groups concealed in the binary of suppression vs. unsuppression but also informs patient management, optimises HIV care delivery (i.e., precision medicine) for patients with the greatest need, and supports triage for health service provision (38–40). In line with previous recommendations (21, 41), we provide evidence for harnessing population VL metrics from observational data to inform actionable knowledge for improved HIV prevention and care.

The greater likelihood of social isolation, loneliness, lack of social support and poor adherence for those unmarried and from lower SES households, respectively represent negative meso-level dynamics which can explain, in large part, the increased risk of virologic failure (42, 43). As previous studies have suggested, socially distanced, withdrawn, depressed individuals not linked to adherence clubs may lack the necessary resilience to engage in continuous HIV care despite the ubiquitous availability of services (14, 44). In our study, those aged younger than 35 years had a higher rebounding risk and were likely unmarried or lacked the stable presence of a life partner, thus warranting scale-up of decentralized services and personalized adherence support for adolescence and young adults as reported elsewhere (45, 46). On the other hand, HIV patients from poor socio-economic backgrounds are known to have less access to public HIV services (47), thus having low rates of sustained viral suppression, as shown in a US study on individuals of varied neighborhood characteristics (48) demonstrating that socio-economic deprivation sustains treatment gaps. Among other factors (49) in limited resource rural settings in sub-Saharan Africa, where healthcare facilities are sparse (i.e., as in the Agincourt HDSS, seven clinics in a 450km^2^ area), patients from wealthier households are likely to afford high travel costs to overcome the largest distance barrier to access services (50) compared to those with lower socio-economic statuses.

Strengths of this study include being among the first to employ Sankey diagrams and this type of transition analysis to characterize different virologic outcome profiles to multi-year HIV VL data. These methods allow for the granular description of the complex VL dynamics in an HIV patient cohort linked to HDSS platform and warrant application in similar settings across sub-Saharan Africa. However, our findings should be interpreted in view of the following limitations. Our study used a facility-based cohort, which would underestimate the true population of the patients engaged in care and the prevalence of viral suppression. For the Agincourt HDSS (outside of the Clinic-Link nested study), population-level blood sample collection for HIV and VL testing commenced in 2020 and may only be available for time-series analyses in the future. Further, the current analysis found that greater than 50% of HIV patients eligible for routine VL testing had no results. This does not only potentially highlight the impact COVID-19 (i.e., government mandates on movement restrictions, social distancing etc.) on access to HIV services (36) and data collection, but overrepresents population groups missing visits in 2020 in this study, which increases the risk selection bias. We did not examine viral outcomes for patients switching to health centres outside the Agincourt HDSS or lost-to-follow-up (LTFU) as discussed elsewhere (51–53) Patients missing scheduled VL testing in clinics, for instance, those that are LTFU, commonly affect HIV surveillance platforms among pregnant or breastfeeding women, those formerly Mozambican, and individuals recently initiating ART (54–56). Additionally, men were underrepresented in our analytic sampling which validates work linking big health data, including high coverage electronic medical records such as the National Health Laboratory Services, which represents >80% of all HIV patients in South Africa, as pioneered elsewhere in the country (57). Moreover, it is possible to suggest that residual confounding affected associations observed due to unmeasured or insufficiently controlled variables in a study that could potentially affect the observed associations.

## Conclusion

In this study we examined viral load trajectories and associated socio-demographic risk factors among adults receiving HIV care in a rural setting in Mpumalanga province, South Africa during the period 2016–2020. Although more than 80% of PLHIV had sustained viral suppression, we identified groups either rebounding, ~2–3% or having ART treatment failure, 5% every year. Our study identified subpopulations failing to accrue the preventive benefit conferred by ART informs patient management routines by prioritising patients with the greatest need, and supports triage for health service provision among younger, male, unmarried and poorer HIV patients. As the third 95 target concerns viral suppression, responses increasing education and awareness regarding HIV prevention and treatment and overcoming social and cultural obstacles to healthcare service access, need to be enhanced. Temporal VL metrics on progress toward reaching high HIV suppression potential in HIV hyperendemic and resource constrained settings are needed to enhance evaluation of interventions and identification of modifiable risk factors to improve individual, population health outcomes and halting onward transmissions by 2030.

## Data Availability

Data from the Agincourt HDSS Clinic-Link-System are available from the corresponding author on valid request. Agincourt HDSS data are available through SAPRIN url http://saprin.mrc.ac.za/ and http://saprindata.samrc.ac.za.

## Footnotes

^1^Two VL measurements per patient 9–15 from the first test were attributed to one year for this analysis despite falling into the subsequent calendar year.
