# Supplementary material for: Viral load dynamics among adults receiving HIV care in rural North-Eastern South Africa, 2015–2020: insights from a population-based record linkage study
Source: Front Public Health. 2025 May 7;13:1551847. doi: 10.3389/fpubh.2025.1551847 (PMC12092226; doi:10.3389/fpubh.2025.1551847)
Supplement: Supplementary file 1 [file Table_1.docx]

Table 1

Table 1: Baseline demographic characteristics of the cohort selection sample, frequencies and percent

| Characteristic | Overall | Frequency | Percent |
| --- | --- | --- | --- |
| Cohort selection sample n (%) | *N*=9 896 | 9 896 | 100.0% |
| Sex |  |  |  |
| Men |  | 2 409 | 24.3% |
| Women |  | 7 487 | 75.7% |
| Age categories |  |  |  |
| 15-24y  25-34y |  | 752  2 667 | 7.6%  26.9% |
| 35-44y |  | 3 152 | 31.6% |
| 45-54y |  | 1 882 | 19.0% |
| 55y+ |  | 1 443 | 14.6% |
| Marital status |  |  |  |
| Married |  | 4 821 | 48.7% |
| Unmarried  Missing |  | 2 613  2 462 | 26.4%  24.9% |
| Highest education |  |  |  |
| Primary |  | 2 259 | 22.8% |
| Secondary |  | 5 065 | 51.2% |
| Tertiary  Missing |  | 209  2 363 | 2.1%  23.8% |
| Household wealth quintile |  |  |  |
| Bottom 20% |  | 1 702 | 17.2% |
| Lower 20% |  | 1 700 | 17.2% |
| Middle 20% |  | 1 673 | 16.9% |
| Higher 20% |  | 1 634 | 16.5% |
| Top 20%  Missing |  | 1 273  1 914 | 12.9%  19.3% |
| Residency status |  |  |  |
| Permanent resident |  | 6 937 | 70.1% |
| Temporary migrant |  | 2 959 | 29.9% |
| History of pregnancy |  |  |  |
| No |  | 9 413 | 95.1% |
| Yes |  | 483 | 4.9% |
| Calendar year  2015  2016  2017  2018  2019  2020  Baseline VL dynamic  Sustained suppression  Achieved suppression  Virologic failure  Viral rebound |  | 3 497  1 970  1 579  1 204  974  672  8 094  704  761  337 | 35.3%  19.9%  16.0%  12.2%  9.8%  6.8%  81.9%  7.1%  7.7%  3.3% |
