# Supplementary material for: Viral load dynamics among adults receiving HIV care in rural North-Eastern South Africa, 2015–2020: insights from a population-based record linkage study
Source: Front Public Health. 2025 May 7;13:1551847. doi: 10.3389/fpubh.2025.1551847 (PMC12092226; doi:10.3389/fpubh.2025.1551847)
Supplement: Supplementary file 2 [file Table_2.docx]

Supplementary table 2: Frequencies and distribution of HIV patients across VL trajectories in the logistic models

|  | **Sustained suppression** | | **Achieved suppression** | **Virologic failure** | **Viral rebound** | | **Total** |
| --- | --- | --- | --- | --- | --- | --- | --- |
| Sex |  | |  |  |  | |  |
| Men | 1895 (78.9%) | | 168 (7.0%) | 247 (10.3%) | 91 (3.8%) | | 2401 (100.0%) |
| Women | 6199 (82.9%) | | 536 (7.2%) | 514 (6.9%) | 228 (3.1%) | | 7477 (100.0%) |
| Age categories | | |  |  | | | |
| 15-24y | 491 (65.5%) | | 85 (11.3%) | 124 (16.5%) | 50 (6.7%) | | 750 (100.0%) |
| 25-34y | 2062 (77.5%) | | 265 (10.0%) | 226 (8.5%) | 107 (4.0%) | | 2660 (100.0%) |
| 35-44y | 2633 (83.6%) | | 185 (5.9%) | 240 (7.6%) | 90 (2.9%) | | 3148 (100.0%) |
| 45-54y | 1644 (87.4%) | | 102 (5.4%) | 92 (4.9%) | 43 (2.3%) | | 1881 (100.0%) |
| 55y+ | 1264 (87.8%) | | 67 (4.7%) | 79 (5.5%) | 29 (2.0%) | | 1439 (100.0%) |
| Marital status | | |  |  |  | |  |
| Unmarried | 2201 (84.3%) | | 166 (6.4%) | 18 (6.9%) | 63 (2.4%) | | 2610 (100.0%) |
| Married | 3869 (80.4%) | | 368 (7.6%) | 412 (8.6%) | 166 (3.5%) | | 4815 (100.0%) |
| Highest education | | | | | | | |
| Primary | 1832 (81.2%) | | 151 (6.7%) | 189 (8.4%) | 84 (3.7%) | | 2256 (100.0%) |
| Secondary | 4152 (82.1% | | 369 (7.3%) | 380 (7.5%) | 156 (3.1%) | | 5057 (100.0%) |
| Tertiary | 173 (82.7%) | | 17 (8.1%) | 12 (5.7%) | 7 (3.4%) | | 209 (100.0%) |
| Household wealth quintile | | | | | | | |
| Bottom 20% | 1338 (78.7%) | | 130 (7.6%) | 167 (9.8%) | 66 (3.9%) | | 1701 (100.0%) |
| Lower 20% | 1404 (82.6%) | | 117 (6.9%) | 132 (7.8%) | 47 (2.8%) | | 1700 (100.0%) |
| Middle 20% | 1388 (83.3%) | | 110 (6.6%) | 125 (7.5%) | 44 (2.6%) | | 1667 (100.0%) |
| Higher 20% | 1341 (82.2%) | | 118 (7.2%) | 130 (8.0%) | 43 (2.6%) | | 1632 (100.0%) |
| Top 20% | 1049 (82.7%) | | 91 (7.2%) | 80 (6.3%) | 48 (3.8%) | | 1268 (100.0%) |
| Residency status | | | | | | | |
| Temporary resident | 2365 (80.0%) | | 235 (8.0%) | 246 (8.3%) | 110 (3.7%) | | 2956 (100.0%) |
| Permanent resident | 5728 (82.8%) | | 468 (6.8%) | 515 (7.4%) | 209 (3.0%) | | 6920 (100.0%) |
| History of pregnancy | | | | | | | |
| No | 7706 (82.0%) | | 665 (7.1%) | 717 (7.6%) | 307 (3.4%) | | 9395 (100.0%) |
| Yes | 388 (80.3%) | | 39 (8.1%) | 44 (9.1%) | 12 (2.5%) | | 483 (100.0%) |
| Calendar year | | | | | | | |
| 2015 | 2927 (83.8%) | 192 (5.5%) | | 284 (8.1%) | 92 (2.6%) | 3495 (100.0%) | |
| 2016 | 1627 (82.6%) | 147 (7.5%) | | 146 (7.4%) | 50 (2.5%) | 1970 (100.0%) | |
| 2017 | 1308 (82.8%) | 116 (7.4%) | | 104 (6.6%) | 51 (3.2%) | 1579 (100.0%) | |
| 2018 | 973 (81.0%) | 93 (7.7%) | | 95 (7.9%) | 40 (3.3%) | 1201 (100.0%) | |
| 2019 | 740 (76.5%) | 95 (9.8%) | | 79 (8.2%) | 53 (5.5%) | 967 (100.0%) | |
| 2020 | 519 (77.9% | 61 (9.2%) | | 53 (8.0%) | 33 (5.0%) | 666 (100.0%) | |
